# Regulation of corticotropin releasing hormone receptor type 1 messenger RNA level in Y-79 retinoblastoma cells: potential implications for human stress response and immune/inflammatory reaction

**DOI:** 10.1155/S0962935196000476

**Published:** 1996-10

**Authors:** N. C. Vamvakopoulos, T. O. Sioutopoulou, Z. Mamuris, P. Marcoulatos, P. C. Avgerinos

**Affiliations:** 1 Department of Biology-Genetics University of Thessaly Medical School 22 Papakyriazi Street Larisa 412 22 Greece; 2 University of Athens Medical School Athens Greece; 3 Department of General Sciences University of Thessaly Volos 38221 Greece; 4 Department of Virology Hellenic Pasteur Institute Athens Greece; 5 1st Department of Internal Medicine ‘Evangelismos’ Hospital Athens Greece

## Abstract

We report the regulation of type 1 receptor mRNA in Y-79 human retinoblastoma cells, grown in the absence or presence of pharmacological levels of phorbol esters, forskolin, glucocorticoids and their combinations. To control for inducibility and for assessing the sensitivity of the Y-79 system to glucocorticoids, corticotropin releasing hormone mRNA levels were measured in parallel. All treatments stimulated corticotropin releasing hormone receptor type 1 gene expression relative to baseline. A weak suppression of corticotropin releasing hormone mRNA level was observed during dexamethasone treatment. The cell line expressed ten-fold excess of receptor to ligand mRNA under basal conditions. The findings predict the presence of functional phorbol ester, cyclic AMP and glucocorticoid response elements in the promoter region of corticotropin releasing hormone receptor type 1 gene and support a potential role for its product during chronic stress and immune/inflammatory reaction.


Mediators of Inflammation, 5, 328–333 (1996)

### Regulation of corticotropin releasing hormone receptor type 1 messenger RNA level in Y-79 retinoblastoma cells: potential implications for human stress response and immune/inflammatory reaction

N. C. Vamvakopoulos,[1,CA] T. O. Sioutopoulou,[2] Z. Mamuris,[3] P. Marcoulatos[1,4] and P. C. Avgerinos[5]

[1]Department of Biology-Genetics, University of Thessaly Medical School, 22 Papakyriazi Street, Larisa 412 22, Greece; [2]University of Athens Medical School, Athens, Greece; [3]Department of General Sciences, University of Thessaly, Volos 38221, Greece; [4]Department of Virology, Hellenic Pasteur Institute, Athens, Greece; [5]1st Department of Internal Medicine, 'Evangelismos' Hospital, Athens, Greece

[CA]Corresponding Author
Fax: (+30) 41 532643

**We report the regulation of type 1 receptor mRNA in Y-79 human retinoblastoma cells, grown in the absence or presence of pharmacological levels of phorbol esters, forskolin, glucocorticoids and their combinations. To control for inducibility and for assessing the sensitivity of the Y-79 system to glucocorticoids, corticotropin releasing hormone mRNA levels were measured in parallel. All treatments stimulated corticotropin releasing hormone receptor type 1 gene expression relative to baseline. A weak suppression of corticotropin releasing hormone mRNA level was observed during dexamethasone treatment. The cell line expressed ten-fold excess of receptor to ligand mRNA under basal conditions. The findings predict the presence of functional phorbol ester, cyclic AMP and glucocorticoid response elements in the promoter region of corticotropin releasing hormone receptor type 1 gene and support a potential role for its product during chronic stress and immune/inflammatory reaction.**

**Key words:** Forskolin, Glucocorticoids, Homeostasis, Human corticotropin releasing hormone receptor, Hypothalamic-pituitary-adrenal axis, Immune/inflammatory reaction, Messenger RNA induction, Phorbol esters, Stress response, Y-79 human retinoblastoma cells

### Introduction

Stress response (the organism's ability for adaptive homeostasis) is a major survival resource and an important permissive factor of primal life properties such as growth, reproduction, evolution and adaptation. In mammals, unexpected stimulation or stress, activates the heat shock protein (hsp) system at the cellular level, and the hypothalamic-pituitary-adrenal (HPA) axis at the level of the whole organism.[1] At the molecular level, these two systems communicate through the functional interaction between hsp90 and glucocorticoid receptor (GR).[2] Glucocorticoids are final effectors of the axis, that bind and activate GRs to exert negative feedback regulation at multiple levels of the axis including the proopiomelanocortin (POMC)[3] and corticotropin releasing hormone receptor (CRHR) type 1 genes[4] in the anterior pituitary corticotroph, and the corticotropin releasing hormone (CRH) gene in the paraventricular nucleus (PVN) of the hypothalamus.[5] The CRH system, including the hormone[6] and urocortin[7] ligands, their two receptor types: type 1[8–11] expressed primarily in the brain,[12] and type 2[13–15] with its two splice variant isoforms; α expressed in limited areas of the brain[14] and β expressed in peripheral tissues including the duodenum, skeletal muscle, epididymis and perivascular cells of the heart,[13,16] and the binding protein (CRHBP),[17] is central coordinator of HPA axis activity and acts in concert with glucocorticoids to maintain whole body homeostasis.

Animal studies revealed highly complex regulation and tissue-specific expression patterns of CRH system components, using a variety of molecular probes.[5,12,16,18–21] To this end, studies with cell lines expressing components of the CRH system, either naturally or following transfection, provide valuable alternatives to the multiparametric complexity inherent in the animal model systems approach and complement molecular analyses of critical regulatory aspects.[22–25]

The human retinoblastoma cell line Y-79, expresses functional CRHRs.[26] We report the effect of forskolin, phorbol esters, dexamethasone and their combinations, on CRHR type 1 mRNA levels in Y-79 cells, using CRH mRNA coexpression as induction control.

© 1996 Rapid Science Publishers

### Materials and Methods

### Cell line and culture conditions

Y-79 is a suspension culture distributed by American Type Culture Collection. The cell line was adopted to growth in steroid-free synthetic media containing antibiotics (CHO-S-SFM, Gibco -BRL Inc.). We assessed that growth under these conditions, does not impair the expression of CRHR. For that, we reacted biotinylated CRH tracer to protein homogenates immobilized onto nitrocellulose filters. Tracer binding was competed with excess CRH but not with dynorphin or growth hormone releasing hormone competitors (data not shown). Binding of biotinylated tracer was recorded on X-ray film by means of a chemiluminescent based detection system (New England Biolabs Inc.). Concentrated cell suspensions were added to 100 × excess of fresh media, and induced by phorbol esters (T; to 100 ng/ml), forskolin (F; to 25 μM), dexamethasone (D; to $10^{-6}$ M) and their combinations to the indicated final concentrations. Following addition of the appropriate inducers, the cell suspensions were incubated at 37°C in 95% $CO_2$–5% air for 3 days. Inductions and controls were performed in triplicate. By analogy to other cell lines,[24,27] these treatments had no toxic effect to the cultured Y-79 cells.

### Preparation of DNA probes

The probe used for CRHR type 1 mRNA hybridization was a 371 bp long fragment of the 3′ non-coding region of the human CRHR type 1 gene that was prepared by direct polymerase chain reaction (PCR) amplification of total human genomic DNA template as described.[28] We used a 785 bp long fragment of CRH cDNA spanning from position 1 (A of the initiator AUG codon) to 785 (in the 3′ non-coding region) according to the numbering system of Shibahara *et al.*,[29] as a hybridization probe for CRH mRNA. This fragment was amplified by PCR in a 50 μl reaction containing 50 ng clone 11 DNA template (human genomic CRH plasmid),[30] 50 nmol of each primer ($hCRH_{f(1-22)}$: 5′-ATGCGGCTGCCGCTGCTTGTGT-3′ and $hCRH_{r(754-785)}$: 5′-GTGTTGCTGCTGCACGTGAATACACTTTGTCG-3′) and Stratagene's DNA polymerase and buffer system, for 30 cycles (denaturation at 94°C for 1 min, annealing at 58°C for 30 s, and extension at 72°C for 1 min). The PCR reaction products were fractionated on agarose gels and the 371 bp CRHR and 785 bp CRH cDNA fragments were gel purified, quantitated, and $P^{32}$-labelled by nick translation, as described.[27] Both probes were labelled to similar specific activities. Preparation and use of human β actin cDNA probe for normalization of Northern data, was as previously described.[27]

### Northern blot analysis

Preparation of total cytoplasmic RNA, was performed as previously described.[27] Signal strength was assessed by direct counting wet filters by means of a horizontal radioactivity counting device (Betascope 603 blot analyser, Betagen, Waltham, MA), as described.[27] All analyses were performed in triplicate and the fold inductions obtained were within 10% from the mean. Representative mean total RNA preparations for every experimental condition, were prepared by combining equal (spectrophotometrically) amounts of RNA from each triplicate point, and this report summarizes the Northern data generated by these pooled samples.

### Materials

The sources of the various reagents used in this study, were as follows: Cells (ATTC, Rockville, MD), α-$^{32}$P-dCTP (3 000 Ci/mmol, ICN, Costa Mesa, CA, USA), random primer nick translation kit (Amersham, Arlington Heights, IL, USA), culture media, antibiotics, ethidium bromide and RNA size standards (Gibco-BRL Life Technologies Inc., Gaithersburg, MD), 12-O-tetradecanoylphorbol 13-acetate (T), forskolin (F) and dexamethasone (D) inducers (Sigma, St Louis, MO), DNA polymerases for PCR amplification (Stratagene, La Jolla, CA and Perkin Elmer-Cetus, Foster City, CA), peptides (Peninsula Labs Inc., Belmont, CA), φX-174 *Hae*III digested DNA size standards and chemiluminescent based detection system (New England Biolabs, Beverly, MA), Kodak XR X-ray film and intensifying screens (Eastman Kodak, Rochester, NY).

### Results

### Induction of CRHR type 1 mRNA

Equal portions of a concentrated suspension of Y-79 cells were diluted 100× with fresh media, the indicated amounts of inducer(s) T, F, D, T + F, T + D, F + D, T + F + D were added, and incubated at 37°C for 3 days. Each induction was performed in triplicate. Following treatment, the cells were collected, washed in PBS, and total RNA was extracted as described.[27] For each condition, triplicate total RNA quantitations were made, yielding minor variations by about 10% from the mean, and equal portions were combined to yield a pooled total RNA sample per condition. Northern analyses of these pooled samples are shown in Figs 1 and 2.

The quality of pooled total RNA from each treatment group, was assessed by pilot agarose gel electrophoresis using 1 μg RNA per lane, along with RNA size standards. A representative preparation is shown in Fig. 1A. The ethidium bromide staining pattern of the eight different RNAs, suggested that the preparations contained primarily intact material.

The degree of induction of CRHR type 1 gene expression, was determined by hybridization of the Northern blotted gel containing 20 μg of total RNA per lane, to a 371 bp long fragment of the 3′ non-coding region of CRHR type 1 DNA that was prepared by direct PCR amplification of human total genomic DNA template.[28] The results are shown in Fig. 1B. All the inducers elevated CRHR type 1 mRNA relative to control. Dexamethasone was the most potent single stimulant, producing a maximal 4.5-fold increase in CRHR type 1 mRNA level alone or in combination with F, and a 3.5-fold increase in CRHR type 1 mRNA when D induction was made in the presence of T and T + F, Fig. 1B.

### Induction of CRH mRNA

The induction of CRH mRNA by forskolin/PKA, phorbol esters/PKC and glucocorticoid pathways has been reviewed.[31] The Y-79 cell system expresses detectable amounts of CRH mRNA by Northern blotting (novel finding of this study). We therefore measured CRH mRNA levels in order to assess the responsiveness of the Y-79 cells to the various treatments as well as the relative levels of receptor and ligand and their responses to common inducers.

CRH mRNA detection, was made on a duplicate blot to that used for CRHR type 1 mRNA analysis. The probe used for hybridization, was a 785 bp DNA segment of the CRH gene including all the protein coding region and a small portion of the 3′ non-coding region of exon 2, that was amplified from clone 11, a genomic clone containing the human CRH gene and its flanking regions,[30] as detailed in the Methods section.

Ethidium bromide staining of the electrophoretic pattern of the PCR reaction product, is shown in Fig. 2A. Co-induction of CRH mRNA level, is shown in Fig. 2B. Long exposures (8 days) were used to detect CRH mRNA signals. A five-fold excess of receptor to ligand cpm was detected in non induced Y-79 cells, by means of the betascope. This difference might be closer to tenfold taking into account the nearly two-fold size difference between CRHR type 1 and CRH cDNA probes used for Northern blotting detection. The effect of inducers and particularly dexamethasone on CRH mRNA levels,

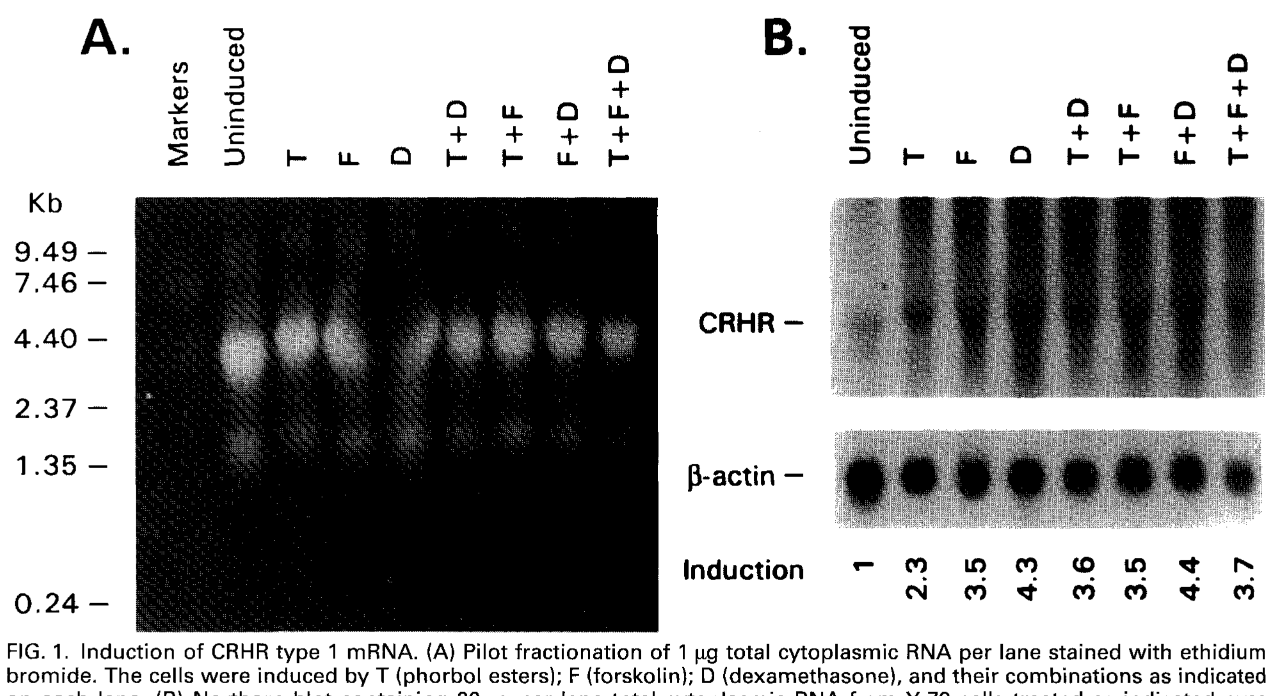

FIG. 1. Induction of CRHR type 1 mRNA. (A) Pilot fractionation of 1 μg total cytoplasmic RNA per lane stained with ethidium bromide. The cells were induced by T (phorbol esters); F (forskolin); D (dexamethasone), and their combinations as indicated on each lane. (B) Northern blot containing 20 μg per lane total cytoplasmic RNA from Y-79 cells treated as indicated, was hybridized to the 371 bp hCRHR-specific DNA type 1 probe that had been prepared by PCR.[28] The blot was stripped and reprobed with an actin probe, and the normalized induction-fold values measured by direct quantitation are shown below each lane.

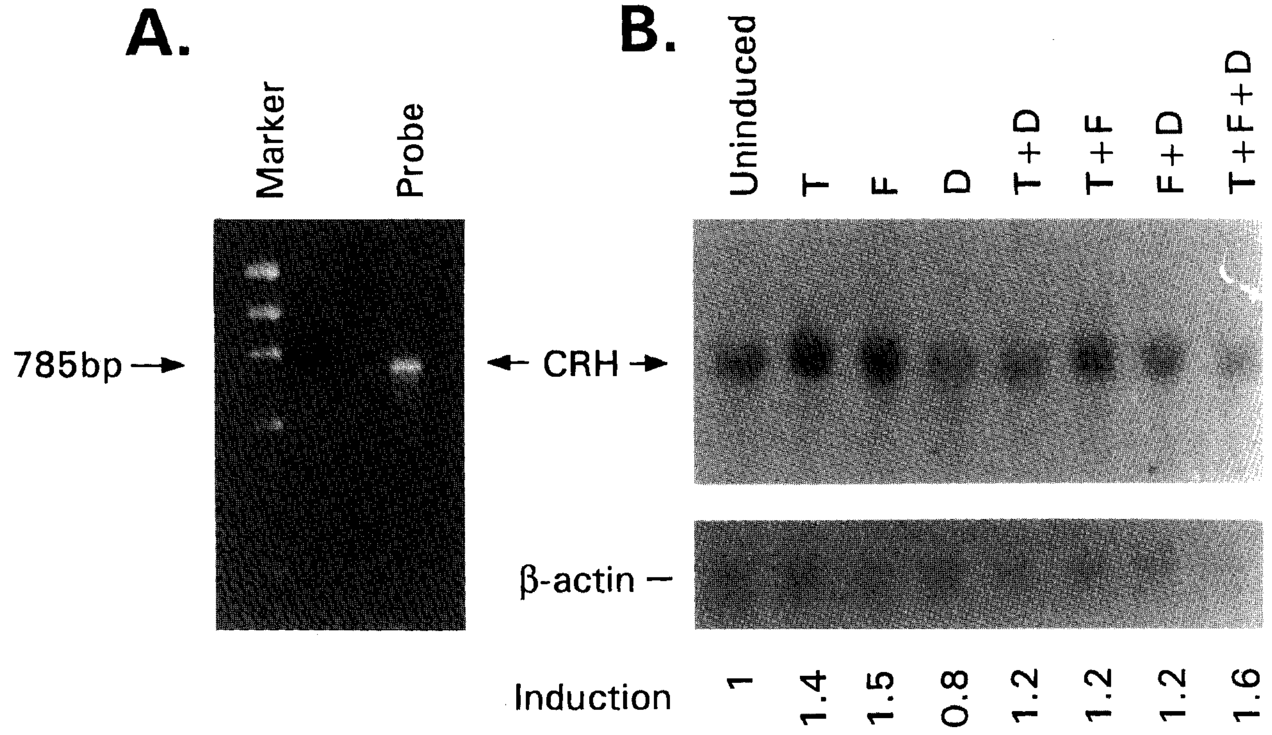

FIG. 2. Induction of CRH mRNA. (A) Electrophoretic pattern of the 785 bp CRH cDNA PCR fragment that was used as a probe, along ϕX-174 *Hae*III DNA size standards. (B) Northern blot containing 20 μg per lane total cytoplasmic RNA from Y-79 cells treated as indicated, was hybridized to the 785 bp long hCRH-specific DNA probe that had been prepared by PCR. The blot was stripped and reprobed with an actin probe. Signal strengths were measured by direct horizontal quantitation of the filters, as described.[27] The normalized induction-fold values are shown below each lane.

suggests that Y-79 cells are of hypothalamic PVN type and not of placental or central nucleus of the amygdala type, tissues where glucocorticoids upregulate CRH gene expression.[5,18–19] This is also supported by the common glucocorticoid upregulation of CRHR type 1 mRNA in rat hypothalami[4,20] and Y-79 cells.

### Discussion

We studied the regulation of CRHR type 1 mRNA in Y-79 human retinoblastoma cells by phorbol esters, forskolin, glucocorticoids and their combinations, using CRH gene coexpression as internal control. Given the comparable processing of the filters shown in Figs 1 and 2, the observed background of the Northern blot of Fig. 1, suggests most probably the detection of additional transcripts with homology to CRHR type 1 mRNA (i.e. CRHR type 2 mRNA with 70% homology,[8] etc.). Although it could be argued that the background may affect the accuracy of the calculated induction values, the observed direction of CRHR type 1 mRNA induction trends remains informative. In the absence of regulatory interference by vasopressin, oxytocin and other neuroendocrine factors, glucocorticoids, suppressed CRH, and stimulated CRHR type 1 gene expression, in the Y-79 environment. In line with HPA axis regulation, glucorticoids, appear to suppress CRH and stimulate CRHR type 1 gene expression in the PVN of rat hypothalamus.[4,19–20] This correlation weakens the possibility of potential cell line dependent regulatory artifacts and predicts that glucocorticoids may also stimulate human hypothalamic CRHR type 1 gene expression. We cannot exclude the possibility that dexamethasone, a well-known apoptotic factor, may have triggered apoptosis in our system, causing induction of gene expression, not necessarily in line with the HPA regulatory system. However, apoptosis of human lymphocytes, known for their enhanced sensitivity to glucocorticoids, was not seen at the level of the hsp90 system under similar culture conditions,[27] excluding indirectly this possibility in the present Y-79 cellular suspension culture system.

In addition to glucocorticoids, forskolin and phorbol esters stimulated both receptor and ligand gene expression, suggesting the presence of functional protein kinase A (PKA) and c(PKC) signal transduction pathways in Y-79 cells, as well as the presence of functional phorbol ester (TRE), cyclic AMP (CRE) and glucocorticoid response elements (GRE) in the promoter region of CRHR type 1 gene. Combined treatments were used to sustain and confirm the overall primary response trends of CRHR type 1-ligand genes in the Y-79 cell system. Maximal induction for the CRHR type 1 gene was five-fold and for the CRH gene two-fold. These differences may reflect either the relative potencies of the response elements between the two genes, or the presence of novel receptor-specific response element(s). Basally, Y-79 express ten-fold excess of receptor to ligand mRNA, suggesting a potentially inverse autonomous regulation of ligand expression by the receptor.[32] We have not determined whether ligand may also regulate receptor expression in this system.

Our finding of CRHR type 1 mRNA up-regulation with concomitant CRH mRNA down-regulation by dexamethasone in the Y-79 human retinoblastoma cell line, underscores the regulatory plasticity of the CRH system for the maintenance and/or restoration of homeostasis. The potential biological significance of this observation may be that during classical hypercortisolaemic states such as pregnancy, depression or Cushing's disease,[33] the selective elevation of CRHR type 1 (i.e. in the hypothalamus,[4,20] or other sites) may increase the sensitivity of the CRH system and prime the organisms homeostatic rebound response.[34] By analogy to immune CRH,[31] local elevation of immune CRHR type 1 in inflammatory sites, such as the arthritic joints of patients with rheumatoid arthritis, may increase the sensitivity of the immune CRH system and prime homeostatic rebound response at the local level. This mechanism, may also account for the beneficial effect of local glucocorticoid administration to inflammatory sites. Further studies on the regulation of CRHR type 2 gene expression by dexamethasone will be needed to support such correlational generalizations.

### References

1. Vamvakopoulos NC, Fukuhara K, Patchev V, Chrousos GP. Effect of single and repeated immobilization stress on the heat shock protein (HSP) 70/90 system of the rat: glucocorticoid independent, reversible reduction of HSP90 in the liver and spleen. *Neuroendocrinology* 1993; **57**: 1057–1065.
2. Picard D, Khursheed B, Garabedian MJ, Fortin MG, Lindquist S, Yamamoto KR. Reduced levels of hsp90 compromise steroid receptor action *in vivo*. *Nature* 1990; **348**: 166–168.
3. Tremblay Y, Tretzakoff I, Peterson A, Autakly T, Zhang CX, Drouin J. Pituitary-specific expression and glucocorticoid regulation of a proopiomelanocortin fusion gene in transgenic mice. *Proc Natl Acad Sci USA* 1988; **85**: 8890–8894.
4. Luo X, Kiss A, Rabadan-Diehl C, Aguilera G. Regulation of hypothalamic and pituitary corticotropin-releasing hormone receptor messenger ribonucleic acid by adrenalectomy and glucocorticoids. *Endocrinology* 1995; **136**: 3877–3883.
5. Swanson LW, Simmons DM. Differential steroid hormone and neuronal influence on peptide and mRNA levels in CRH cells of the paraventricular nucleus: a hybridization histochemical study in the rat. *J Comp Neurol* 1989; **285**: 413–435.
6. Vale W, Spiess J, Rivier C, Rivier J. Characterization of a 41-residue ovine hypothalamic peptide that stimulates secretion of corticotropin and β-endorphin. *Science* 1981; **213**: 1394–1397.
7. Vaughan J, Donaldson C, Bittencourt J, Perrin MH, Lewis K, Sutton S, Chan R, Turnbull AV, Lovejoy D, Rivier C, Rivier J, Sawchenko PE, Vale W. Urocortin, a mammalian neuropeptide related to fish urotensin I and to corticotropin-releasing factor. *Nature* 1996; **378**: 287–292.
8. Chen R, Lewis KA, Perrin MH, Vale WW. Expression cloning of a human corticotropin-releasing-factor receptor. *Proc Natl Acad Sci USA* 1993; **90**: 8967–8971.
9. Vita N, Laurent P, Lefort S, Chalon P, Lelias J-M, Kaghad M, Le Fur G, Ferrera P. Primary structure and functional expression of mouse pituitary and human brain corticotrophin releasing factor receptors. *FEBS Lett* 1993; **335**: 1–5.
10. Chang CP, Pearse RI, O'Connell S, Rosenfeld MG. Identification of a seven transmembrane helix receptor for corticotropin releasing factor and sauvagine in mammalian brain. *Neuron* 1993; **11**: 1187–1195.
11. Perrin MH, Donaldson CJ, Chen R, Lewis KA, Vale WW. Cloning and functional expression of a rat brain corticotropin releasing factor (CRF) receptor. *Endocrinology* 1993; **133**: 3058–3061.
12. Potter E, Sutton S, Donaldson C, Chen R, Perrin M, Lewis K, Sawchenko P, Vale W. Distribution of corticotropin-releasing factor receptor expression in rat brain and pituitary. *Proc Natl Acad Sci USA* 1994; **91**: 8777–8781.
13. Perrin MH, Donaldson CJ, Chen R, Blount A, Berggren T, Bilezikjian L, Sawchenko P, Vale WW. Identification of a second CRF receptor gene and characterization of a cDNA expressed in heart. *Proc Natl Acad Sci USA* 1995; **92**: 2969–2973.
14. Lovenberg TW, Liaw CW, Grigoriadis DE, Clevenger W, Chalmers DT, De Souza EB, Oltersfdorf T. Cloning and characterization of a functionally distinct corticotropin-releasing factor receptor subtype from rat brain. *Proc Natl Acad Sci USA* 1995; **92**: 836–840.
15. Kishimoto T, Pearse RV, Lin CR, Rosenfeld MG. A sauvagine/corticotropin-releasing factor receptor expressed in heart and skeletal muscle. *Proc Natl Acad Sci USA* 1995; **92**: 1108–1112.
16. Lovenberg TW, Chalmers DT, Liu C, De Souza EB. $CRF_{2\alpha}$ and $CRF_{2\beta}$ receptor mRNAs are differentially distributed between the rat central nervous system and peripheral tissues. *Endocrinology* 1995; **136**: 4139–4142.
17. Potter E, Behan DP, Fischer WH, Linton EA, Lowry PJ, Vale WW. Cloning and characterization of the cDNAs for human and rat corticotropin-releasing factor-binding proteins. *Nature* 1991; **349**: 423–426.
18. Robinson BG, Emanuel RL, Frim DM, Majzoub JA. Glucocorticoid stimulates expression of corticotropin-releasing hormone gene in human placenta. *Proc Natl Acad Sci USA* 1988; **85**: 5244–5248.
19. Makino S, Gold PW, Schulkin J. Corticosterone effects on corticotropin-releasing hormone mRNA in the central nucleus of the amygdala and the parvocellular region of the paraventricular nucleus of the hypothalamus. *Brain Res* 1994; **640**: 105–112.
20. Luo X, Kiss A, Makara G, Lolait SJ, Aguilera G. Stress-specific regulation of corticotropin releasing hormone receptor expression in the paraventricular and supraoptic nuclei of the hypothalamus in the rat. *J Neuroendocrinol* 1994; **6**: 689–696.
21. Kiss A, Palkovits M, Aquilera G. Neural regulation of corticotropin releasing hormone (CRH) and CRH receptor mRNA in the hypothalamic paraventricular nucleus in the rat. *J Neuroendocrinol* 1996; **8**: 103–112.
22. Dorin RI, Takahashi H, Nakai Y, Fukata J, Naitoh Y, Imura H. Regulation of human corticotropin-releasing hormone gene expression by 3′,5′-cyclic adenosine monophosphate in a transformed mouse corticotroph cell line. *Mol Endocrinol* 1989; **3**: 1537–1544.
23. Adler GK, Rosen LB, Fiandaca MJ, Majzoub JA. Protein kinase-C activation increases the quantity and poly(A) tail length of corticotropin-releasing hormone messenger RNA in NPLC cells. *Mol Endocrinol* 1992; **6**: 476–484.
24. Vamvakopoulos NC, Chrousos GP. Regulated activity of the distal promoter-like element of the human corticotropin releasing hormone gene and secondary structural features of its corresponding transcripts. *Mol Cell Endocrinol* 1993; **94**: 73–78.
25. Vamvakopoulos NC, Chrousos GP. Evidence of direct estrogenic regulation of human corticotropin releasing hormone gene expression: potential implications for the sexual dimorphism of the stress response and immune/inflammatory reaction. *J Clin Invest* 1993; **92**: 1896–1902.
26. Olianas MC, Lampis G, Onali P. Human Y-79 retinoblastoma cells express functional corticotropin-releasing hormone receptors. *Brain Res* 1992; **593**: 304–306.
27. Vamvakopoulos NC, Mayol V, Margioris A, Chrousos GP. Lack of dexamethasone modulation of mRNAs involved in the glucocorticoid signal transduction pathway in two cell systems. *Steroids* 1992; **57**: 282–287.
28. Vamvakopoulos NC, Sioutopoulou TO. Human corticotropin releasing hormone receptor gene (CRHR) is located on the long arm of chromosome 17 (17q12-qter). *Chromosome Res* 1994; **2**: 471–473.
29. Shibahara S, Morimoto Y, Furutani Y, Notake M, Takahashi H, Shimizu

S, Numa S. Isolation and sequence analysis of the human corticotropin-releasing factor precursor gene. *EMBO J* 1983; **2**: 775–779.
30. Vamvakopoulos NC, Chrousos GP. Structural organization of the 5′ flanking region of the human corticotropin releasing hormone gene. *DNA Sequence J DNA Sequencing and Mapping* 1993; **4**: 197–206.
31. Vamvakopoulos NC. Sexual dimorphism of stress response and immune/inflammatory reaction: the corticotropin releasing hormone perspective. *Mediators Inflamm* 1995; **4**: 163–174.
32. Milano CA, Allen LF, Rockman HA, Dolber PC, McMinn TR, Chien KR, Johnson TD, Bond RA, Lefkowitz RJ. Enhanced myocardial function in transgenic mice overexpressing the $\beta_2$-adrenergic receptor. *Science* 1994; **264**: 582–586.
33. Orth DN. Corticotropin-releasing hormone in humans. *Endocrine Rev* 1992; **13**: 164–191.
34. Michelson D, Gold PW. Stress responsive neurohormones in depression and anxiety. In: denBoer JA, Ad Stzen JM, eds. *Handbook of Depression and Anxiety: a biological approach*. Marcel Dekker, 1994; 529–542.

ACKNOWLEDGEMENTS. We thank Dr I. Maroulacou for helpful discussion, G. Poy for oligo-DNA syntheses and B. Alexander for assistance with the Northern procedure.

**Received 26 June 1996; accepted in revised form 16 August 1996**
